# Atomic Modulation of 3D Conductive Frameworks Boost Performance of MnO_2_ for Coaxial Fiber-Shaped Supercapacitors

**DOI:** 10.1007/s40820-020-00529-8

**Published:** 2020-10-27

**Authors:** Xiaona Wang, Zhenyu Zhou, Zhijian Sun, Jinho Hah, Yagang Yao, Kyoung-Sik Moon, Jiangtao Di, Qingwen Li, Ching-ping Wong

**Affiliations:** 1 Division of Advanced Nanomaterials, Key Laboratory of Nanodevices and Applications, Joint Key Laboratory of Functional Nanomaterials and Devices, CAS Center for Excellence in Nanoscience, Suzhou Institute of Nano-Tech and Nano-Bionics, Chinese Academy of Sciences, Suzhou, 215123 People’s Republic of China; 2 School of Materials Science and Engineering, Georgia Institute of Technology, Atlanta, GA 30332 USA

**Keywords:** Coaxial fiber-shaped supercapacitors, 3D framework, Zn–CuO nanowires, Zn–CuO@MnO_2_ core–shell structure

## Abstract

Coaxial fiber-shaped supercapacitors are a promising class of energy storage devices requiring high performance for flexible and miniature electronic devices. Yet, they are still struggling from inferior energy density, which comes from the limited choices in materials and structure used. Here, Zn-doped CuO nanowires were designed as 3D framework for aligned distributing high mass loading of MnO_2_ nanosheets. Zn could be introduced into the CuO crystal lattice to tune the covalency character and thus improve charge transport. The Zn–CuO@MnO_2_ as positive electrode obtained superior performance without sacrificing its areal and gravimetric capacitances with the increasing of mass loading of MnO_2_ due to 3D Zn–CuO framework enabling efficient electron transport. A novel category of free-standing asymmetric coaxial fiber-shaped supercapacitor based on Zn_0.11_CuO@MnO_2_ core electrode possesses superior specific capacitance and enhanced cell potential window. This asymmetric coaxial structure provides superior performance including higher capacity and better stability under deformation because of sufficient contact between the electrodes and electrolyte. Based on these advantages, the as-prepared asymmetric coaxial fiber-shaped supercapacitor exhibits a high specific capacitance of 296.6 mF cm^−2^ and energy density of 133.47 μWh cm^−2^. In addition, its capacitance retention reaches 76.57% after bending 10,000 times, which demonstrates as-prepared device’s excellent flexibility and long-term cycling stability.

**Electronic supplementary material:**

The online version of this article (10.1007/s40820-020-00529-8) contains supplementary material, which is available to authorized users.

## Highlights

- 3D Zn-doped CuO framework was designed for aligned distributing high mass loading of MnO_2_ nanosheets.
- Zn could be introduced into the CuO crystal lattice to tune the covalency character and thus improve charge transport.
- A free-standing asymmetric coaxial fiber-shaped supercapacitor based on Zn–CuO@MnO_2_ core electrode possesses superior performance including higher capacity and better stability under deformation.

**Electronic supplementary material:**

The online version of this article (10.1007/s40820-020-00529-8) contains supplementary material, which is available to authorized users.

## Graphical abstract

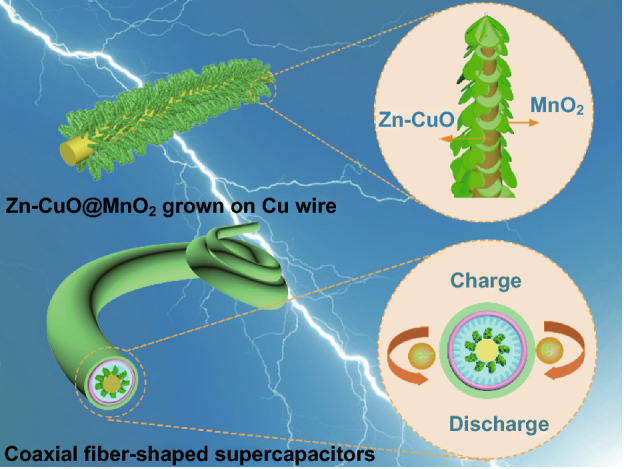

## Introduction

The multifunctional portable, wearable devices are a mainstream direction in electronics field and have attracted interesting attention in recent years [1]. These devices, including electrochemical supercapacitors, rechargeable secondary batteries, artificial skin sensors have been widely studied [2–4]. However, the conventional planar devices are not suitable for the application of flexible and wearable electronics. Fiber-shaped supercapacitors have a vital position as a new class of flexible power source because of lightweight, small volume, facility in the device application and integration in wearable electronics [5]. Nevertheless, their practical applications are still limited severely since inferior energy density is related to the narrow potential window and low capacitance. Therefore, substantial efforts have been made to improve the energy density via widening the overall potential voltage (V) and improvement of specific capacitance (C) [6].

Fabrication of asymmetric fiber-shaped supercapacitors (AFSC) is an effective strategy to enlarge the voltage window by assembling of two different types of electrode materials [5]. However, AFSC is comprised of two parallel fiber electrodes; it is difficult to realize free-standing because these fibers need to be placed onto substrates [7]. A twisted-type fiber supercapacitor was presented using plastic wire, polymer Kevlar fiber or carbon fiber, but, the effective connective surface area of the two electrodes was limited resulting in low specific capacitance, low energy density, as well as leakage current risk between electrodes during bending or stretchable process [8–10]. The asymmetric coaxial fiber-shaped supercapacitor (ACFSC) was designed by assembling two electrodes into a core–shell electrode structure with a fiber electrode core, an film electrode shell and an electrolyte material sandwiched in-between [11]. This coaxial structure provides superior performance including higher capacity and better stability under deformation because of sufficient contact between the electrodes and electrolyte. However, it is still a challenge to coat uniform films on a fiber core and retain the electrochemical stability under deformation.

Furthermore, besides the configuration of the electrodes, the active materials play the important role in improving the electrochemical performance of ACFSC. Among the rich pool of various pseudocapacitive materials, manganese oxide (MnO_2_) has attracted tremendous attention for its high theoretical specific capacitance, low cost, non-toxicity, wide availability, and environmental friendliness [12, 13]. Unfortunately, its inferior electrical conductivity (10^–5^ to 10^–6^ S cm^−1^) leading to limited specific capacitance and power capability generates an adverse influence in development of ACFSC with excellent electrochemical properties [14]. The prompting studies were boosted to fabricate MnO_2_ composite electrodes containing graphene, carbon nanotubes, and so on [15, 16]. However, probably because of the severe aggregation of active materials leading to the increasing of “dead” mass which unavoidably hinder the electron transfer, unsatisfied energy density still exists in the carbon/MnO_2_ hybrid composite structure as ACFSC electrode materials. In addition, the thin film structure or a small active material loading (0.1–0.8 mg cm^−2^) on the substrates is generally required to achieve satisfactory electrochemical properties because of the redox reaction occurring only in a few nanometers of electrode surface [13]. With the increasing MnO_2_ mass loading, the capacitive performance was found to deteriorate severely due to slow ion diffusion and inefficient electron transport. Thus, the cycling stability and rate capacity are also impacted seriously resulting in hindering the further application as ACFSC electrode materials. A typical MnO_2_ mass loading of 8–10 mg cm^−2^ is required to provide necessary capacitance for commercial SC [17]. Deposition of MnO_2_ onto three-dimensional conductive scaffold, including carbon, metal oxide or metal nanomaterials, has been proven to be an effective way to obtain larger electroactive surface area and the short diffusion routes for electrons and ions in the electrolyte [18–22]. Carbon nanomaterials, such as carbon nanotube, graphene and porous carbon, were usually used as 3D conductive skeletons which displays the superior electrochemical performance owing to the smart hybrid structure and synergistic effect [23, 24]. However, probably because of the mixture of conductive additive or polymer binder leading to the increasing of “dead” mass which unavoidably hinder the electron transfer, unsatisfied energy density still exists in the carbon/metal oxide/hydroxide hybrid composite structure as fiber-shaped electrode. Metal nanomaterials with high electrical conductivity have been developed as 3D conductive support such as porous gold, copper nanowires, porous Ni, but there is almost no capacitance contributing during electrochemical charge and discharge process [20, 21]. However, there are few reports to design a 3D scaffold with high performance for homogeneous distribution of active materials toward free-standing hybrid electrode for ACFSC.

Herein, we report the rational design and growth of Zn–CuO@MnO_2_ core–shell nanowire arrays as high-performance positive electrode for ACFSC. In situ Zn-doped CuO nanowires on Cu wire were one-step grown and designed as skeletons for aligned distributing MnO_2_ nanosheets. The electrical conductivity of Zn doped into the lattice of CuO was improved by several magnitude compared with pristine CuO nanowires, which enables efficient electron transport in the redox reactions of MnO_2_ nanosheets. Meanwhile, Zn-doped CuO also contributes electrochemical capacitance for MnO_2_ composite electrode. Zn_0.11_CuO with MnO_2_ loading of 12.4 mg cm^−2^ achieves a high areal capacitance of 4.26 F cm^−2^ (33.2 F g^−1^). Zn_0.11_CuO@MnO_2_ as core positive electrode could be assessed as a new category of free-standing ACFSC positive electrode associated with high specific capacitance and stability cycling performance. Vanadium nitride (VN) nanowire arrays grown on carbon nanotubes film (CNF) were wrapped uniformly on the core positive electrode as negative electrode to form the coaxial construction devices. The as-prepared ACFSC exhibits a maximum operating voltage window of 1.8 V, a high specific capacitance of 107.95 F cm^−3^ (296.6 mF cm^−2^), energy density of 133.5 mWh cm^−2^ (at a power density of 0.9 mW cm^−2^) and power density of 8.9 mW cm^−2^ (at an energy density of 68.7 mWh cm^−2^), which demonstrates as-prepared device could be a promising candidate for potential uses in high-performance ACFSC.

## Experimental Section

### Synthesis of Zn–CuO@MnO_2_ Core–Shell Nanowires on Cu Wires

First, copper wires were cleaned using ethanol and acetone solution with ultrasound for 10 min, respectively. Copper wires were annealed at 600 °C for 30 min in Ar to remove the oxide layer on the surface of copper. The samples were then immersed into Zn(CH_3_COO)_2_ aqueous solutions with varied concentration (0.05 ~ 1 M). Then, the samples were put into oven at 60 °C for 30 min to evaporate the moisture. Next, the samples were heated in air from room temperature to 500 °C with 2 °C min^−1^ and kept at 500 °C for 4 h in tube furnace to grow the Zn-doped CuO nanowires. CuO nanowires grown onto Cu wires were fabricated by the same method without using Zn(CH_3_COO)_2_ solutions. MnO_2_ nanosheets were grown onto the Zn-doped CuO/Cu nanowires via a facile electrodeposition method in a mixed aqueous solution of 0.05 M MnSO_4_, 0.05 M CH_3_COONa, and 10% vol ethanol with a current density of 5 mA cm^−2^.

### Fabrication of All-Solid-State ACFSC Device

VN nanowires array was synthesized on CNT film by our previous method [25]. The Na_2_SO_4_/PVA sol–gel electrolyte was firstly prepared by mixing 10 g of PVA and 10 g of Na_2_SO_4_ into 100 mL of deionized water under vigorous stirring at 85 °C for 2 h. The Zn–CuO@MnO_2_ core–shell nanowires on Cu wires electrode were soaked into the Na_2_SO_4_/PVA sol–gel electrolyte for 5 min and then maintained at 60 °C for 2 h to evaporate excess moisture. After the gel electrolyte was dried, the aligned VN@CNT film was wrapped around core electrode. The area of the VN@CNT film could be easily adjusted by changing the helical angle. The gel electrolyte was then coated onto the outside of ACFSC.

### Characterization

Scanning electron microscopy (SEM) was carried out using Hitachi S–4800 with an accelerating voltage of 3 kV. X-ray diffraction (XRD) patterns were obtained using a Rigaku D/MAX2500 V with Cu Kα radiation (λ = 1.5418 Å). X-ray photoelectron spectroscopy (XPS) patterns were acquired an ESCALab MKII X-ray photoelectron spectrometer with non-monochromatized Mg Kα X-rays as the excitation source. Inductively coupled plasma emission spectrometer (ICP) data were obtained by Agilent 5100 to confirm the mass of Zn-doped CuO lattice. High-resolution transmission electron microscopy (HRTEM) and energy dispersive X-ray (EDX) elemental mapping images were acquired by a FEI TECNAI G2 20 high resolution TEM (200 kV). Electrochemical measurements were taken on the CHI 760e electrochemical working station.

## Results and Discussion

**Fig. 1:**
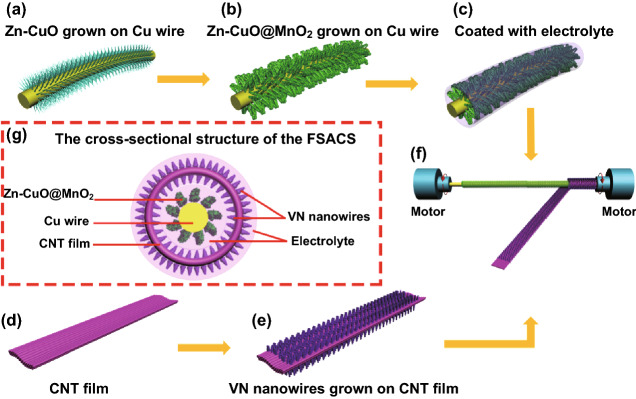
**a–f** Schematic illustrations of the fabrication of the ACFSC. **g** Cross-sectional structure of the ACFSC

The fabrication process of the ACFSC device is schematically displayed in Fig. 1. First, Zn–CuO nanowires were fabricated by annealing Cu wire covering Zn ions in air in tube furnace (Fig. 1a). Then, MnO_2_ nanosheets were grown onto the Zn–CuO/Cu wire via a facile electrodeposition method in a mixed aqueous solution of MnSO_4_, CH_3_COONa, and ethanol with a current density of 5 mA cm^−2^ (Fig. 1b). The positive core electrode was coated with a thin layer of poly(vinyl alcohol) (PVA) gel electrolyte including Na_2_SO_4_ as gel electrolyte and separator (Fig. 1c). VN nanowire arrays were synthesized on the CNT film as negative electrode shown in Fig. 1e. Afterward, it was wrapped around the positive electrode (inner core electrode) whose two ends were immobilized on two motors while the motors were rotating (Fig. 1f). Finally, the second layer of PVA gel electrolyte was coated on the outside of ACFSC. The cross-profile of ACFSC device is illustrated in Fig. 1g, demonstrating that the aligned structure probably favored the rapid charge transport and diffusion of electrolyte ions.

The X-ray diffraction (XRD) patterns of the as-prepared CuO and Zn–CuO with different molar concentration of dopant are displayed in Fig. S1. The pattern of pure CuO phase shows a typical diffraction peaks at 2θ of 35.85, 38.96, 48.89, 61.56, 66.38, and 68.16, corresponding to ($\overline{1}11$), (200), ($\overline{2}02$), ($\overline{1}13$), ($\overline{3}11$), and (220) reflection planes of monoclinic crystalline CuO. The pattern of Zn–CuO nanowires shows that there is no trace of Zn or its compounds, indicating dopants are well integrated into CuO lattice structure during the growth process. The peaks of Zn–CuO are found to be wider than those of CuO, indicating the existence of asymmetry crystalline structure after doping. Meanwhile, the position of peaks slightly shifted toward higher angles for Zn_0.05_CuO, Zn_0.08_CuO, Zn_0.11_CuO and slightly shifted toward lower angles with further increasing of Zn dopants for Zn_0.15_CuO, Zn_0.28_CuO because Zn substitution generates residual stress that might result in anisotropic shrinkage of lattices and then induce lattice distortion. Tensile stress brings about diffraction planes shift of lower angle, while pressure stress can cause the shift to higher angles [26]. The electric conductive properties of ZnCuO were improved greatly compared with pure CuO. The sample of Zn_0.11_CuO shows the superior electrical conductivity of compared with other samples (Fig. S2). Therefore, Zn_0.11_CuO was chosen as conductive skeleton to support the deposition of MnO_2_ nanosheets. The chemical compositions and valence states of the Zn_0.11_CuO@MnO_2_ were investigated using XRD patterns. Compared with the XRD pattern of Zn_0.11_CuO nanowires, new peaks at 12.8°, 60.2^o^ are attributed to MnO_2_ (JCPDF No. 44–0141) (Fig. S3).

**Fig. 2:**
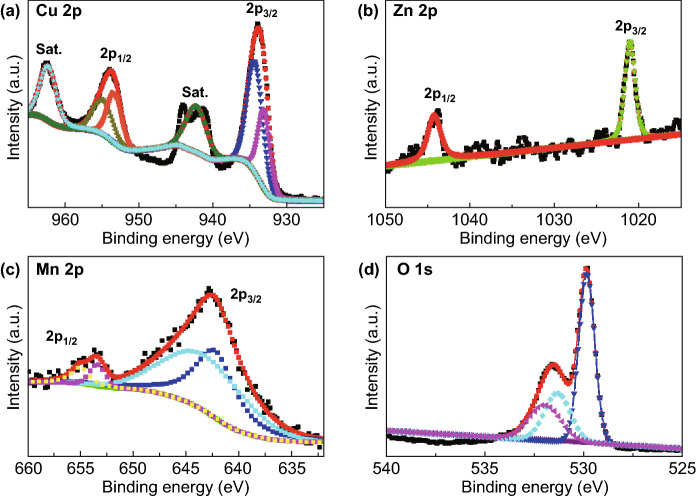
**a** Cu 2*p,* **b** Zn 2*p*, **c** Mn 2*p* and **d** O 1*s* regions for Zn–CuO@MnO_2_ core–shell nanowire array structure

The XPS data of CuO and Zn–CuO are displayed in Fig. S4. It is found that the intensity of O 1*s* of 531.5 eV binding energy of Zn–CuO is more than that of CuO sample, demonstrating oxygen vacancies increase after Zn doping into CuO. The main reason is probably that enhances pseudocapacitive charge storage properties of Zn–CuO. The detailed analysis is exhibited in the supporting information. The XPS peaks of Cu 2*p* at 934.5 and 954.2 eV are assigned to Cu 2*p*_3/2_ and Cu 2*p*_1/2_, illustrating the presence of the Cu^2+^ for Zn_0.11_CuO@MnO_2_ sample (Fig. 2a) [27–29]. The XPS spectrum of Zn 2*p* displays a peak centered at 1044.21 and 1021.15 eV that is attributed to Zn 2*p*_1/2_ and Zn 2*p*_3/2_, demonstrating that is present Zn^2+^ (Fig. 2b) [30]. The Mn 2*p* XPS spectrum reveals Mn 2*p*_3/2_ peak Mn 2*p*_1/2_ peak at binding energy of 642.6 and 653.4 eV, indicating the presence of Mn^4+^ (Fig. 2c) [17, 31]. The O 1*s* binding energies of 529.8 and 531.5 eV are the main components typical for oxygen in Zn_0.11_CuO@MnO_2_ (Fig. 2d).

**Fig. 3:**
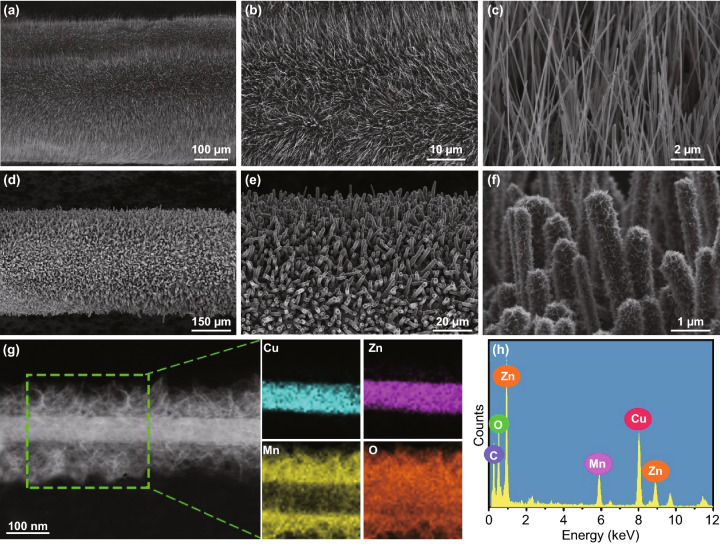
**a–c** SEM images of CuO nanowire array grown on Cu wire at different magnifications. **d–f** SEM images of the MnO_2_ nanosheets coating on Zn–CuO nanowire array to form core–shell nanowire structure. **g** TEM image of a single Zn–CuO@MnO_2_ core–shell nanowire structure and X-ray elemental mappings of different elements of Cu, Zn, Mn, and O recorded from an individual core–shell nanowire structure. **h** X-ray elemental spectrum of the Zn–CuO@MnO_2_

The morphology and crystalline structure of the CuO nanowire, Zn_0.11_CuO nanowires and Zn_0.11_CuO@MnO_2_ core–shell heterostructure were characterized by SEM and TEM. The different magnification SEM images of the Zn_0.11_CuO nanowire arrays on the surface of Cu wire in Fig. 3a–c reveal that the uniformly distributed nanowire arrays on the surface of Cu wire when heated at 500 °C for 4 h. The average diameter of Zn_0.11_CuO nanowire arrays is 100 nm, and lengths are from 100 to 200 nm. The SEM images of MnO_2_ nanosheets deposited onto the Zn_0.11_CuO nanowires are shown in Fig. 3d–f. It is found that the plenty of uniform and ultrathin MnO_2_ nanosheets are aligned distributed on the surface of ZnCuO nanowires, forming the perfect 3D configuration with a reasonable space between the adjacent nanowire arrays. The thickness of MnO_2_ layer on the Zn_0.11_CuO increases with the electrodeposition time, as shown in Fig. S5. TEM images provide further insight into detailed structure of CuO, Zn_0.11_CuO and Zn_0.11_CuO@MnO_2_ heterostructure nanowire arrays shown in Fig. S6. Figure 2g clearly displays that internal nanowire structure with the diameter of ~ 80 nm is covered by the interconnected tiny nanosheets. These ultrathin nanosheets increase the specific surface area of electrode materials. EDX elemental mapping images totally confirm the hierarchical core–shell structure in which Cu, Zn, and O are uniformly distributed in the internal structure and Mn and O are distributed in the external structure. The EDX spectrum in Fig. 2h shows several elements such as Cu, Zn, Mn, and O appeared in the hierarchical structure without any impurities. The C is believed from the TEM grid with ultrathin carbon film. These results illustrate the significance of using ZnCuO as skeletons in achieving a uniform coating of MnO_2_ at high mass loading and verify the successful integration of 3D highly conductive ZnCuO and MnO_2_ ultrathin nanosheets.

**Fig. 4:**
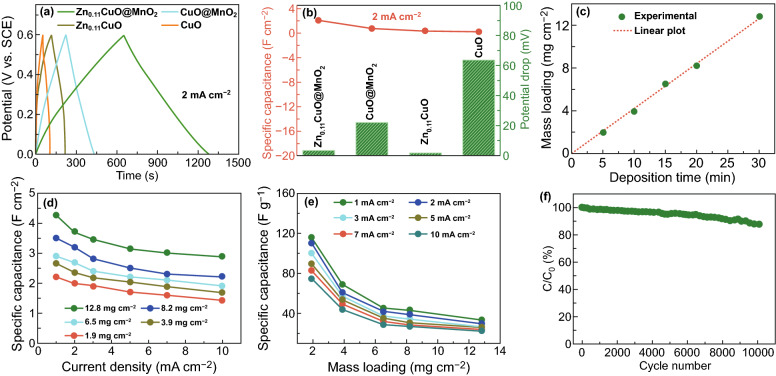
**a** Comparison of GCD curves of typical CuO, CuO@MnO_2_, Zn_0.11_CuO and Zn_0.11_CuO@MnO_2_ electrodes at a current density of 2 mA cm^−2^. **b** Areal capacitances and potential drops of obtained from CuO, CuO@MnO_2_, Zn_0.11_CuO, and Zn_0.11_CuO@MnO_2_ electrodes. **c** Relationship between the mass loading of MnO_2_ and the electrodeposition time. **d, e** Areal capacitances and gravimetric capacitances of Zn_0.11_CuO@MnO_2_ electrodes with different amount of MnO_2_ at different current densities. **f** Capacitance retention plotted versus cycle number

The electrochemical properties of electrodes were investigated in a three-electrode system in 1 M Na_2_SO_4_ aqueous electrolyte. The effect of the content of Zn on the capacitive performance of Zn–CuO electrodes is shown in Fig. S7. It is observed that there is no obvious trend for capacitance change of Zn–doped CuO electrodes with increasing contents of Zn. The detailed analysis and charge transfer mechanism are described in Supporting Information. The galvanostatic charge–discharge (GCD) curves of CuO, Zn_0.11_CuO, CuO@MnO_2_ and Zn_0.11_CuO@MnO_2_ electrodes at a current density of 2 mA cm^−2^ over a potential range from 0 to 0.6 V are shown in Fig. 4a. Zn_0.11_CuO nanowires electrode has the longer discharge time than pure CuO, illustrating Zn doping into CuO could effectively reduce the overall resistance and improve electron collection rates and the charge transport during electrochemical reaction. When the deposition time of MnO_2_ on Zn–CuO is just 5 min with a loading of 1.9 mg cm^−2^, the discharge time of electrode is significantly increased compared to CuO, Zn–CuO, CuO@MnO_2_. The specific capacitance was calculated, and the corresponding results of these electrodes are plotted in Fig. 4b, according to Eq. S1. The areal capacitance of Zn_0.11_CuO@MnO_2_ electrodes is 2.2 F cm^−2^ which is larger than that of CuO, Zn_0.11_CuO, even CuO@MnO_2_ electrodes at current density of 2 mA cm^−2^. The internal resistances (IR) of these electrodes are associated with the electrical conductivity of materials, which is proportional to the potential drop of charge and discharge curves. The potential drop of Zn_0.11_CuO electrode is about 0.0018 V, which is more 30 times lower than that of the CuO electrode (the potential drop is 0.0636 V) at a current density of 2 mA cm^−2^. Although the potential drop of core–shell electrode is increased slightly after the deposition of MnO_2_ due to its poor electrical conductivity, it is still much less than CuO@MnO_2_ electrodes. These results illustrate that ion diffusion and charge transfer in these 3D Zn_0.11_CuO@MnO_2_ core–shell electrodes are highly rapid and efficient during charge and discharge process. Different mass loading of MnO_2_ nanosheets on the Zn_0.11_CuO was studied by varying deposition time of 5, 10, 15, 20, and 30 min (Fig. S8). It allows precise control of mass loading of MnO_2_ ultrathin nanosheets on 3D Zn–CuO skeleton, because it is linear relationship between deposition time and mass loading of MnO_2_ (Fig. 4c). The high loading mass of 12.4 mg cm^−2^ MnO_2_ nanosheets was obtained after 30 min deposition. With the increasing of MnO_2_ loading, the areal capacitance of the Zn_0.11_CuO@MnO_2_ electrode also increases at different current densities (Fig. 4d). The capacitance of Zn_0.11_CuO@MnO_2_ electrode with the loading mass of 12.4 mg at current density of 10 mA cm^−2^ (2.88 F cm^−2^) corresponds to 68% capacitance retention relative to that of 1 mA cm^−2^, which indicates the superior rate capability of hierarchical 3D core–shell electrode. And when the loading mass of MnO_2_ increased from 1.9 to 12.4 mg cm^−2^, the gravimetric capacitances of Zn_0.11_CuO@MnO_2_ electrode decrease slightly (Fig. 4e). Therefore, this advanced 3D Zn_0.11_CuO@MnO_2_ core–shell electrode supports the long-term cyclic sustainability (Fig. 4f). The 87.6% capacitance of the 3D core–shell electrode is retained after 10,000 cycles at a current density of 10 mA cm^−2^. When the deposition time of MnO_2_ was further increased, it rendered to touch each other between the adjacent nanowires to form a dense block area and reducing the effective contactable surface area leading to inferior electrochemical performance; corresponding SEM images and electrochemical properties are shown in Fig. S9. The electrochemical impedance spectroscopy (EIS) studies were conducted and are shown in Fig. S10. These results illustrate that Zn_0.11_CuO@MnO_2_ possesses the lowest charge-transfer and series resistances which contribute to its large capacitance and good rate capability.

**Fig. 5:**
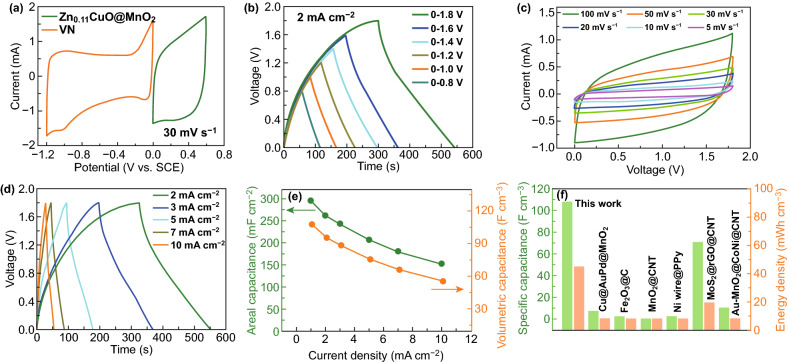
**a** Comparison of CV curves of the Zn_0.11_CuO@MnO_2_ positive electrode and VN@CNT film negative electrode. **b** GCD curves of ACFSC device collected over different voltages from 0.8 to 1.8 V at a current density of 2 mA cm^−2^. **c** CV curves of ACFSC device at various scan rates. **d** GCD curves of ACFSC device at different current densities. **e** Comparison of areal and volumetric specific capacitance curves of ACFSC device calculated from the charge–discharge curves as a function of the current density. **f** Areal specific capacitances and energy densities of our device in comparison with previously reported ACFSC

Then, the ACFSC was successfully assembled by wrapping the VN on CNT film around the positive electrode (inner electrode), which is displayed in Figs. S11 and S12 by corresponding low- and high-magnification SEM images. The potential windows of the VN/CNT film and Zn_0.11_CuO@MnO_2_ electrodes were − 1.2 to − 0.2 V and 0 to 0.6 V, respectively (Fig. 5a). Therefore, the maximum voltages window for the asymmetric supercapacitor could reach 1.8 V. The GCD curves were also tested at a current density of 2 mA cm^−2^ with different potential window from 0.4 to 1.8 V and displayed nearly triangular shape at a potential window as high as 1.8 V, indicating the ideal electrochemical capacitance characteristics and reversible Faradaic reaction (Fig. 5b). A series of CV curves for the asymmetric coaxial device was measured at various scan rates from 5 to 100 mV s^−^ (Fig. 5c) and reveal quasi-rectangular shape of CV shapes without obvious redox peaks when the potential increases to 1.8 V, implying good high-rate performance. The GCD curves of the ACFSC device were measured between 0 and 1.8 V for the current densities from 2 to 10 mA cm^−2^ (Fig. 5d). These curves are linear that illustrates which this coaxial fiber-shaped device displays ideal capacitive properties and low equivalent series resistance. The areal and volumetric capacitances of as-fabricated ACFSC device were calculated and plotted as a function of current density from 1 to 10 mA cm^−2^ (Fig. 5e). It is worth noting that the device can achieve a very high volumetric capacitance of 107.9 F cm^−3^ (296.6 mF cm^−2^) at a current density of 1 mA cm^−2^ and 55.6 mF cm^−3^ (152.7 F cm^−2^) at 10 mA cm^−2^, indicating its excellent rate performance. For ACFSC device, the energy density (*E*) and power density (*P*) which are two important parameters for evaluating the electrochemical performance, were calculated from GCD curves and plotted on the Ragone diagram (Fig. 5f). Notably, with the volumetric capacitance of 107.95 F cm^−3^, our ACFSC device illustrates a high volumetric energy density of 48.53 mWh cm^−3^ and volumetric power density of 327.2 mW cm^−3^ obtained. These values are much greater than those of some recently reported FSC devices, such as MoS_2_@rGO@CNT [32], Au-MnO_2_@CoNi@CNT [33], Ni wire@PPy [34], MnO_2_@CNT [35], Fe_2_O_3_@C [36], and Cu@AuPd@MnO_2_ [37]. A Ragone plot, representing the relationship between the energy density and power density of the ACFSC device, is shown in Fig. S13, illustrating a high areal energy density of 133.5 µWh cm^−2^ and an areal power density of 0.9 mW cm^−2^ obtained at a current density of 2 mA cm^−2^. These values are much higher than those of some recently reported FSC devices and MnO_2_ supercapacitors, such as CNT@MnO_2_ AFSC [32], CNT@NiO@MnO*x* FSC [38], nickel–cobalt layered double hydroxide FSC [39], carbon fiber@MnO_2_ supercapacitors [40], carbon fabric@MnO_2_ micro-supercapacitor [41], zinc–nickel–cobalt ternary oxides AFSC [42], MnO_2_@PEDOT:PSS@CNT FSC [43], conductive polymer@RuO_2_ FSC [44], (PEDOT:PSS)/PPy FSC [45], and MnO_2_ NBs@Ni/CNT coaxial FSC [46].

**Fig. 6:**
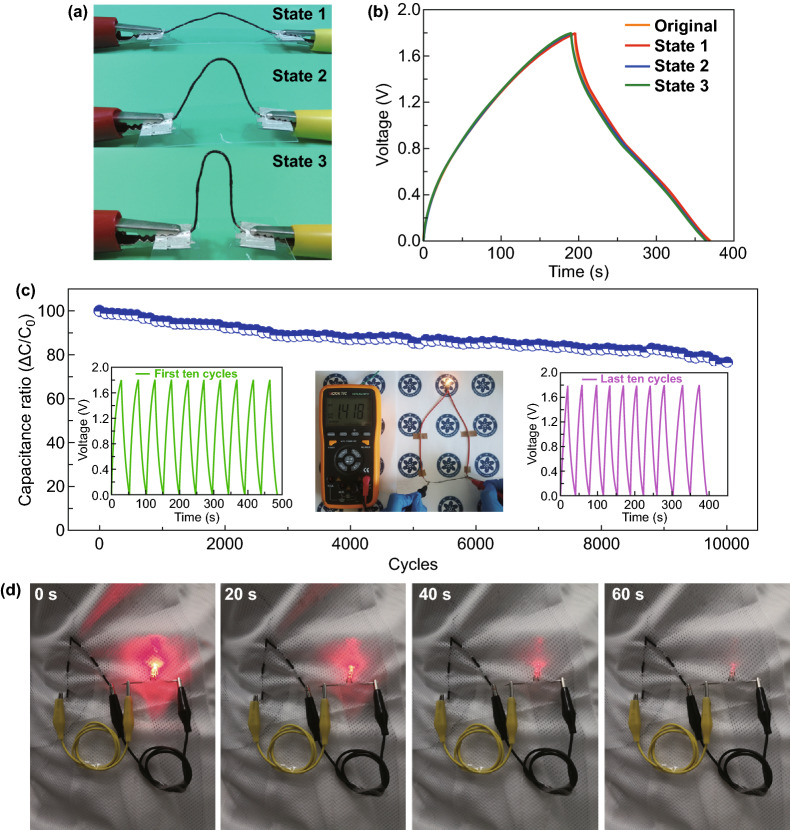
**a** Optical images of an ACFSC at different bending state. **b** GCD curves obtained at different bending states corresponding to images in **a**. **c** Cycle stability tested at 90° bending states. Inset are the charge–discharge curves at the first ten cycles and the last ten cycles. The middle image shows that the ACFSC can power a LED light and the voltage of device is 1.418 V. **d** ACFSC powering LED, showing its potential applicability for wearable applications

To further explore their application in flexible electronics, the mechanical stability of the ACFSC devices was investigated under different bending states as shown in Fig. 6a. It is worth noting that all the GCD curves observed at various bending states are no obvious distortion, indicating that the ACFSC device has excellent mechanical stability (Fig. 6b). The long-term cyclic stability and durability of the ACFSC device were further confirmed and are shown in Fig. 6c. It displays that as-prepared ACFSC device retains 76.57% of the initial capacitance after 10,000 charge and discharge cycles at the current density of 10 mA cm^−2^, which illustrates the excellent bending cycle stability of our ACFSC devices. The capacitances of device gradually decrease because the electrode materials are destroyed after several electrochemical redox reactions. A yellow light-emitting diode (LED) is powered by ACFSC device shown in inset of Fig. 6c, indicating the feasibility and potential application of the device. Accordingly, two full charged ACFSC devices woven into a cloth to light the LED, shown in Fig. 6d; it can give the light continuously for 60 s. These results illustrate that ACFSC device using Zn_0.11_CuO@MnO_2_ and VN/CNT film as positive and negative electrodes with high energy storage and good flexible could be applied in wearable electronics as an advanced power source.

## Conclusions

In summary, we designed Zn–CuO nanowire arrays as 3D skeletons for the coating of MnO_2_ as core positive electrode. After Zn doped into the lattice of CuO, the electrical conductivity of Zn–CuO nanowire arrays is increased which provides the rapid route for electron transfer during electrochemical charge and discharge process. Benefiting from the nanowire arrays structure of Zn–CuO, it allows the superior high mass loading pseudocapacitive material without decreasing the gravimetric and areal capacitance. Furthermore, VN nanorods fabricated on CNT film were wrapped onto the core positive electrode to form an asymmetric coaxial ACFSC, which increases the connective surface area and decreases the contact resistance between two electrodes. Also, it could be assessed as a new category of free-standing ACFSC electrode associated with high specific capacitance and stability cycling performance. The as-prepared ACFSC exhibits a high specific capacitance of 107.9 F cm^−3^ (296.6 mF cm^−2^) and energy density of 48.53 mWh cm^−3^. In addition, its capacitance retention reaches 88.45% after bending 3000 cycles, which demonstrates excellent flexibility of our prepared device. With the advantage of excellent energy storage performance and flexibility, the ACFSC device designed in this paper is expected to be integrated into wearable electronics systems.

## Electronic supplementary material

Below is the link to the electronic supplementary material.

Supplementary file1 (PDF 1352 kb)
