# Supplementary material for: Atomic Modulation of 3D Conductive Frameworks Boost Performance of MnO2 for Coaxial Fiber-Shaped Supercapacitors
Source: Nanomicro Lett. 2020 Oct 27;13:4. doi: 10.1007/s40820-020-00529-8 (PMC8187521; doi:10.1007/s40820-020-00529-8)
Supplement: Supplementary file 1 — Supplementary file1 (PDF 1352 kb) [file 40820_2020_529_MOESM1_ESM.pdf]

Supporting Information for

Atomic Modulation 3D Conductive Frameworks Boosts Performance of MnO_2 for Coaxial Fiber-Shaped Supercapacitors

Xiaona Wang[1, 2, *], Zhenyu Zhou[1], Zhijian Sun[2], Jinho Hah[2], Yagang Yao[1], Kyoung-Sik Moon[2], Jiangtao Di[1, *], Qingwen Li[1, *], Ching-ping Wong[2, *]

[1]Division of Advanced Nanomaterials, Key Laboratory of Nanodevices and Applications, Joint Key Laboratory of Functional Nanomaterials and Devices, CAS Center for Excellence in Nanoscience, Suzhou Institute of Nano-tech and Nano-bionics, Chinese Academy of Sciences, Suzhou 215123, People's Republic of China

[2]School of Materials Science and Engineering, Georgia Institute of Technology, Atlanta, Georgia 30332, United States

*Corresponding authors. E-mail: xnwang2016@sinano.ac.cn (Xiaona Wang); jidi2009@sinano.ac.cn (Jiangtao Di); qwli2007@sinano.ac.cn (Qingwen Li); cp.wong@mse.gatech.edu (Ching-ping Wong)

S1 XRD and Electrical Measurement

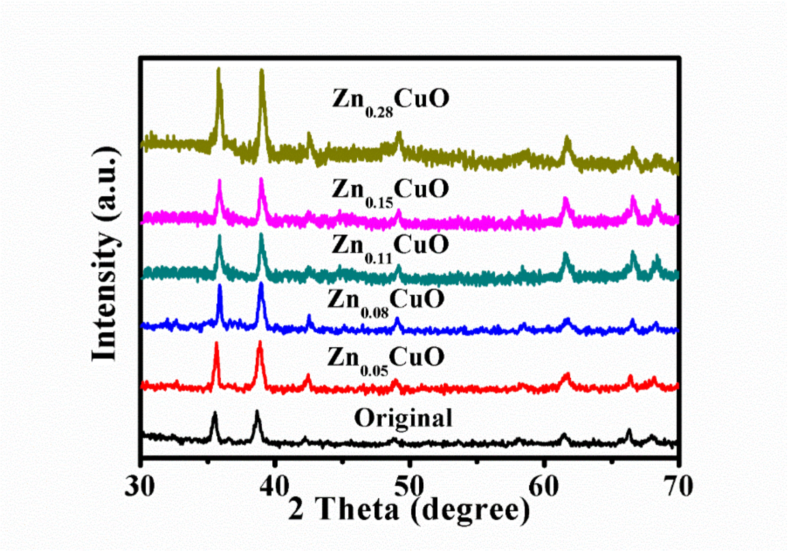

Fig. S1 XRD analysis of CuO and ZnCuO with different molar concentration of dopant

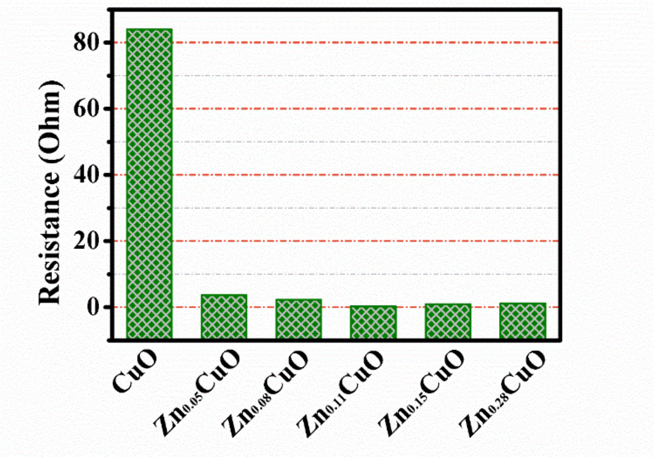

Fig. S2 Resistance of different Zn doping amounts into CuO nanowires

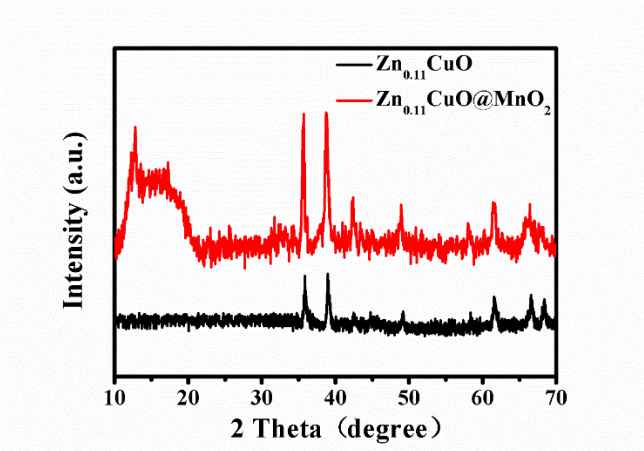

Fig. S3 XRD spectra of $Zn_{0.11}CuO$ and $Zn_{0.11}CuO@MnO_2$

S2 XPS data of CuO and Zn-CuO Samples

The binding energies of 934.5 eV and 954.2 eV are assigned to Cu $2p_{3/2}$ and Cu $2p_{1/2}$ for pristine CuO nanowires, in good agreement with Cu 2p state (Fig. S4b). The detailed XPS pattern of the O1s are fitted with two peaks (Fig. S4c). The binding energies at 529.8 eV can be ascribed to O^{2-} ion present in CuO and higher binding energy (O_2) at 531.5 eV can be attributed to O_2^- and O^- ions in oxygen-deficient regions. High-resolution spectra of the Cu 2p for Zn-CuO sample in Fig. S4d displays similar results with Cu^{2+} of CuO nanowires. The XPS of Zn 2p spectrum fitted with two Gaussian Lorentz peaks shows a peak centered at 1022 eV which is ascribed to Zn $2p_{3/2}$. The binding energy at 1044 eV can be fitted with two Gaussian Lorentz peaks that is attributed to Zn $2p_{1/2}$ (Fig. S4e). This demonstrated that the Zn phases were present in the samples. The intensity of O 1s of 531.5 eV binding energy of Zn-CuO is more than that of CuO sample, demonstrating oxygen vacancies increase after Zn doping into CuO [S1, S2].

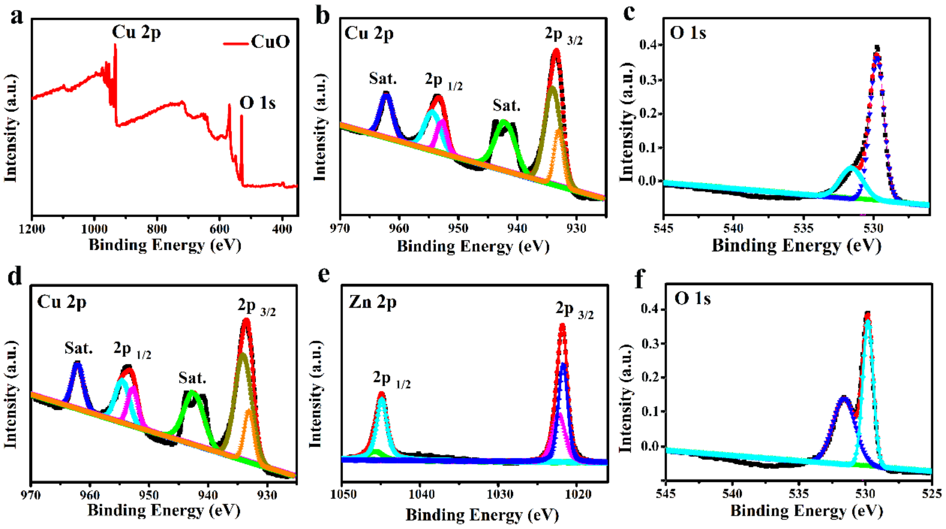

Fig. S4 (**a**) XPS pattern of CuO nanowires. Cu 2p (**b**) and O 1s (**c**) of CuO nanowires. Cu 2p (**d**), Zn 2p (**e**) and O 1s (**f**) of ZnCuO nanowires

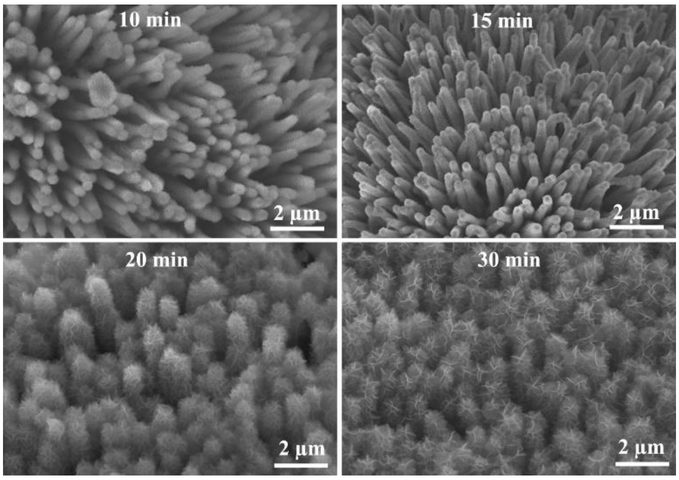

Fig. S5 SEM images of $Zn_{0.11}CuO@MnO_2$ with different deposition times of 10, 15, 20, and 30 min

S3 TEM Characterization

Figure S6a-c show the TEM, HRTEM and SAED images of CuO nanowires produced by heating in air at 500 °C for 4 h. The HRTEM image of the pure CuO nanowires consisted of crystals with a lattice fringe spacing of 0. 275 nm, consistent with the (110) plane of the monoclinic structure of CuO (Fig. S6b). The SAED image of CuO nanowires correspond to the pure CuO nanowires. The CuO nanowires were doped by an in-situ doping mechanism, where doping takes place at the same time that the nanowires are formed. Zn-CuO nanowires are found that growing along the (110) direction, as shown in Fig. S6c-f. The diffraction pattern, shown in Fig. Sf, indicates there is no structural change because of the dopants, which is consistent with XRD data. Fig. S6g-i shows TEM, HRTEM and SAED images of the Zn-CuO nanowires

after MnO_2 deposition, exhibiting that internal nanowire structure with the diameter of ~200 nm is covered by the interconnected tiny nanosheets (Fig. S6g). The HRTEM image of consisted of crystals with a lattice fringe spacing of 0.69 nm and 0.239 nm, consistent with the (110) and (211) planes of the structure of MnO_2. Moreover, the ring pattern spots observed in the SAED confirms polycrystalline nature of the prepared material.

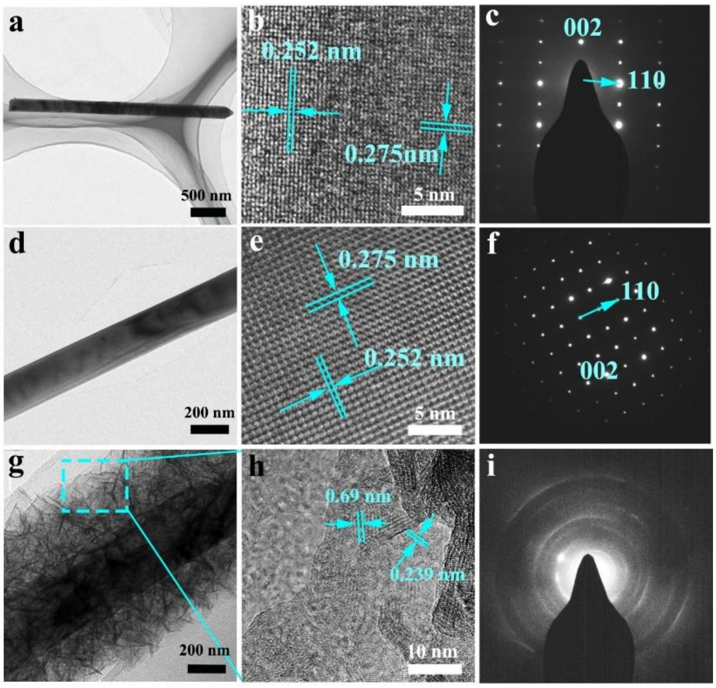

Fig. S6 TEM images of CuO nanowires (**a**), Zn-CuO nanowires (**d**) and Zn-CuO@MnO_2 core-shell nanowires (**g**). High-resolution TEM images of CuO nanowires (**b**), Zn-CuO nanowires (**e**) and Zn-CuO@MnO_2 core-shell nanowires (**h**). Electron diffraction pattern recorded from an individual CuO nanowires (**c**), Zn-CuO nanowires (**f**) and Zn-CuO@MnO_2 core-shell nanowires (**i**)

S4 Electrochemical Performance Measurements

All related electrochemical data including cyclic voltammetry (CV), galvanostatic charge-discharge (GCD) curves were measured by utilizing an electrochemical workstation (CHI 760E, Chenhua). The CuO, Zn-CuO, CuO@MnO_2 and $Zn_{0.11}$CuO@ MnO_2 and VN/CNT film were directly used as the working electrode. A saturated calomel electrode and a platinum electrode were used as the reference electrode and the counter electrode, respectively. The electrochemical properties of the as-fabricated electrodes were characterized by three-electrode system in a 1 M Na_2SO_4 aqueous electrolyte. The electrochemical characterization of all-solid asymmetric coaxial fiber-shaped supercapacitor (ACFSC) device was carried out in a two-electrode system. The specific capacitance was calculated by Eq. S1:

$$C_{cell} = \frac{i \times t}{S \times \Delta V} \qquad \text{(S1)}$$

where C_{cell} is the specific capacitance of the electrode, i is the discharge current during the charge and discharge process, t is the discharge time from high to low potential, S is the area/volume of the electrode, ΔV is the gap of high and low potential windows, respectively.

The energy density (E) and power density (P) were obtained based on Eq. S2 and S3, respectively:

$$E = \frac{C_{cell} \times \Delta V^2}{2} \quad (S2)$$

$$P = \frac{E}{t} \quad (S3)$$

where C_{cell} is the specific capacitance of the electrode, ΔV is the voltage gap of high and low potential windows, t is the discharge time from high to low potential, respectively.

The galvanostatic charge-discharge (GCD) curves of CuO electrodes with the different content of Zn were performed in a three-electrode system in 1M Na_2SO_4 aqueous electrolyte to analyze the effect of the contents of Zn on the capacitive performance. As shown in Fig. S7a, Zn-doped CuO electrodes have a longer discharge time than that of CuO electrode, illustrating Zn doping into CuO have a limited improvement for the capacitance performance of CuO electrode. The specific capacitance was calculated and the corresponding results of these electrodes are plotted in Fig. S7b. It is observed that there is no obvious trend for capacitance change of Zn-doped CuO electrodes with increasing contents of Zn. But, the potential drop of Zn-doped CuO electrodes have a significant decrease, demonstrating Zn doping into CuO could effectively improve the conductivity of CuO electrode, as well as improve electron collection rates and the charge transport during electrochemical reaction.

The charge transfer mechanisms and electrode kinetics were further investigated by Dunn's method, which provides the quantitative separation of the capacitive charge process (electrical double-layer effect and faradaic charge contribution) and diffusion process (capacitance arising from slow ion de/intercalation in the active materials). To obtain the ratio of as-prepared electrodes capacitive contribution from the total capacity, the formula was divided into two parts quantitatively: $i(v) = k_1 v + k_2 v^{1/2}$ (k_1 and k_2 are scan rate independent constants; v *is* scan sweep; $k_1 v$ represents capacity contribution; $k_2 v^{1/2}$ represents diffusion contribution) [S3, S4]. For our Zn-CuO electrodes, their capacitive-controlled capacitances are higher than that of CuO electrodes which illustrates a faster faradaic charge transfer process in Zn-CuO electrodes. $Zn_{0.11}CuO$ and $Zn_{0.15}CuO$ nanowires electrodes possess the larger capacitive-controlled capacitance of about 75% than CuO and other Zn-CuO electrodes (Fig. S7c). Furthermore, we also investigated the charge transfer mechanism of $Zn_{0.11}CuO@MnO_2$ electrode (Fig. S7d). The capacity contribution increases with the increasing of the scan rate. At a scan rate of 100 mV/s, the capacitive-controlled capacitance of the $Zn_{0.11}CuO@MnO_2$ electrode is estimated at 72.6%, indicating that the rapid faradaic charge transfer process onto the surface or

near surface of electrodes.

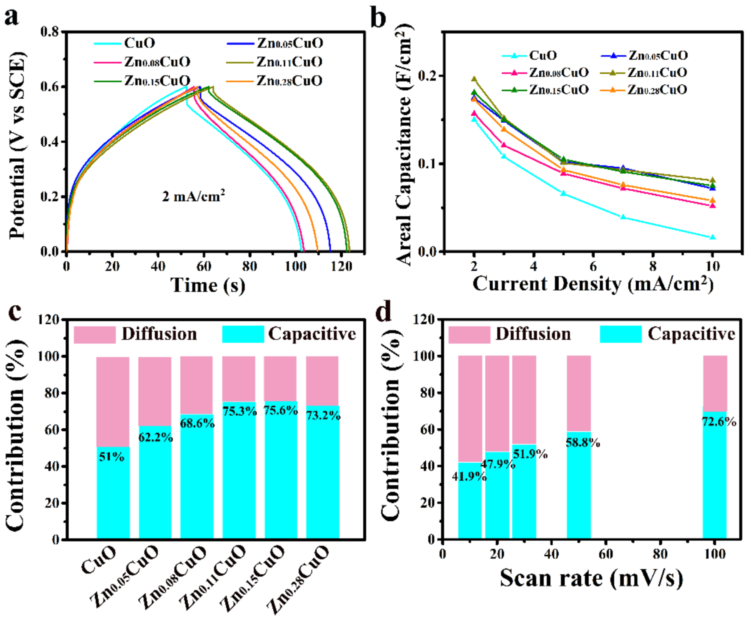

Fig. S7 (**a**) Comparison of GCD curves of typical CuO, $Zn_{0.05}CuO$, $Zn_{0.08}CuO$, $Zn_{0.11}CuO$, $Zn_{0.15}CuO$, $Zn_{0.28}CuO$ electrodes at a current density of 2 mA cm^{-2}. (**b**) Areal capacitances of CuO electrodes with different contents of Zn at different current densities. Histogram illustration of capacitive and diffusive capacitance contribution of CuO and Zn-CuO electrodes at a scan rate of 10 mV s^{-1} (**c**) and $Zn_{0.11}CuO@MnO_2$ electrode at different scan rate (**d**).

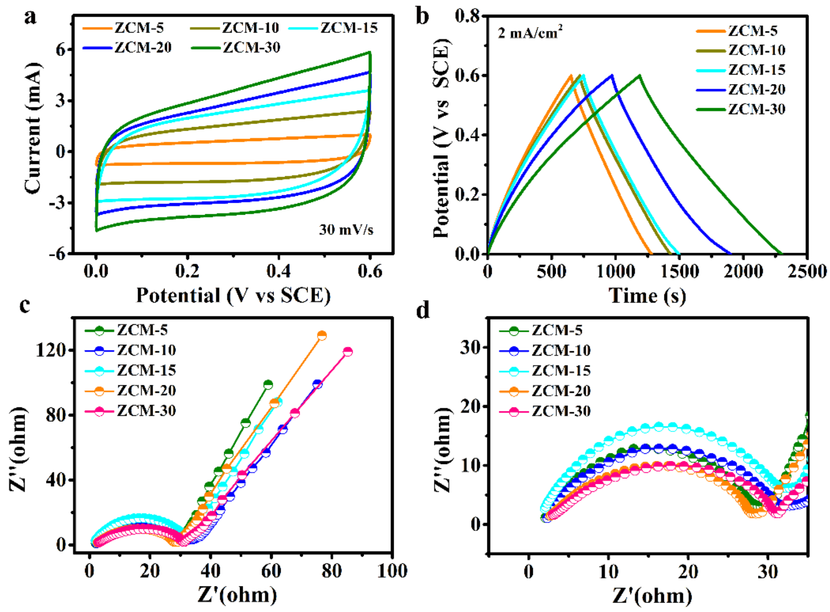

Fig. S8 Electrochemical performance of ZnCuO@MnO2 (ZCM) with different MnO2 deposition time (ZCM-5: 5 min deposition; ZCM-10: 10 min deposition; ZCM-15: 15 min deposition; ZCM-20: 20 min deposition; ZCM-30: 30 min deposition;). CV (**a**), GCD (**b**), EIS (**c**) and enlarge EIS (**d**) curves of ZCM electrodes

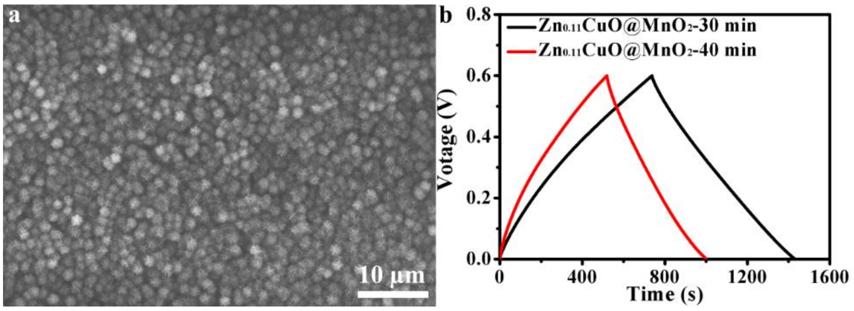

Fig. S9 (**a**) SEM image of $Zn_{0.11}CuO@MnO_2$ with deposition times of 40 min; (**b**) Comparison of GCD curves of $Zn_{0.11}CuO@MnO_2$ with different deposition time of 30 min and 40 min

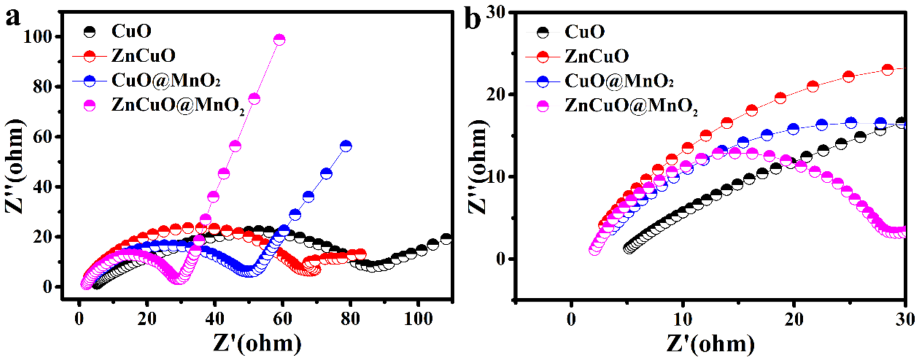

Fig. S10 Nyquist plots (**a**) and high-frequency domain (**b**) collected at open-circuit potential of CuO, ZnCuO, $CuO@MnO_2$ and $ZnCuO@MnO_2$ electrodes

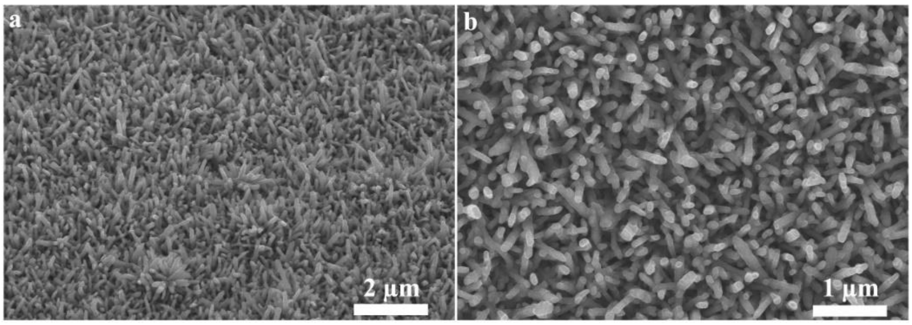

Fig. S11 SEM images of VN nanowires grown on the CNT film

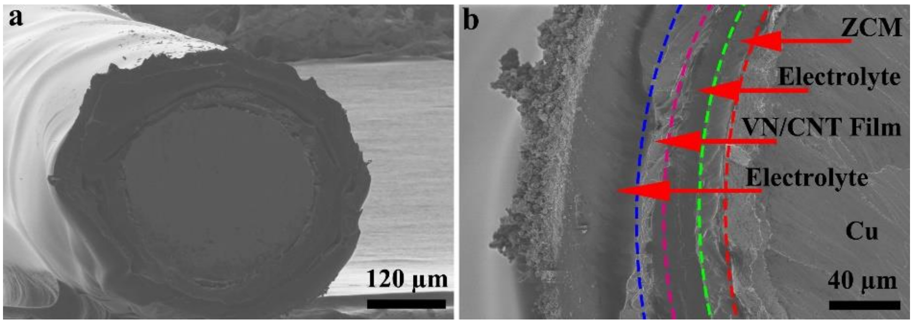

Fig. S12 Low (**a**) and high magnifications (**b**) of SEM images of ACFSC

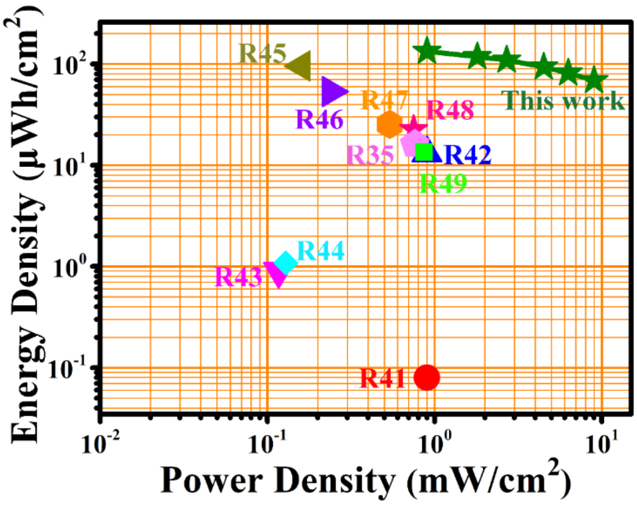

Fig. S13 Energy and power densities of our device in comparison with previously reported literatures

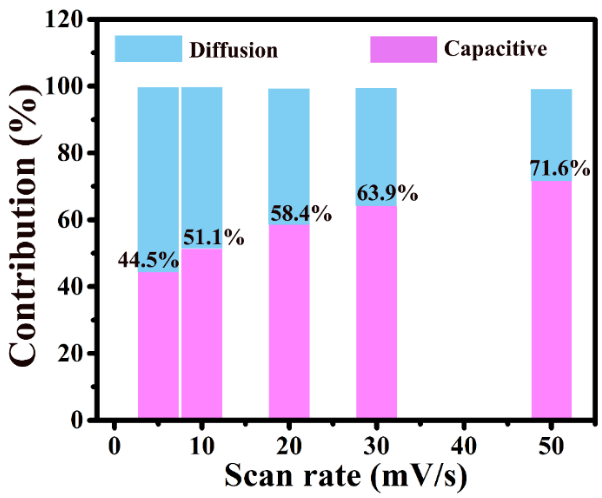

Fig. S14 Histogram illustration of capacitive and diffusive capacitance contribution of as-prepared ACFSC at different scan rate

Supplementary References

[S1] A.K. VJyoti, G.D. Srivastava, Varma, Highly selective and efficient room temperature NO_2 gas sensors based on Zn-doped CuO nanostructure-rGO hybrid. J. Mater. Sci. **29**, 10640 (2018). https://doi.org/10.1007/s10854-018-9128-7

[S2] M. Huang, Y. Zhang, F. Li, Z. Wang, Alamusi, N. Hu, Z. Wen, Q. Liu. Merging of kirkendall growth and ostwald ripening: CuO@MnO_2 core-shell architectures for asymmetric supercapacitors. Sci. Rep. **4**, 4518 (2014). https://doi.org/10.1038/srep04518

[S3] X. Hui, R. Zhao, P. Zhang, C. Li, C. Wang, L. Yin, Low-temperature reduction strategy synthesized Si/Ti_3C_2 MXene composite anodes for high-performance Li-ion batteries. Adv. Energy Mater. **9**, 1901065 (2019). https://doi.org/10.1002/aenm.201901065

[S4] T. Brezesinski, J. Wang, S. H. Tolbert, B. Dunn, Ordered mesoporous α-MoO_3 with iso-oriented nanocrystalline walls for thin-film pseudocapacitors. Nat. Mater. **9**, 146(2010). https://doi.org/10.1038/nmat2612
